# PKD1-mediated phosphorylation at dopamine D2 receptor serine 365 site in dorsal striatum underlies cocaine-induced locomotor hyperactivity

**DOI:** 10.1016/j.ibneur.2025.06.013

**Published:** 2025-06-19

**Authors:** Xinyu Zhang, Ziran Zhang, Ying Wang, Linlin Sun, Ning Wang

**Affiliations:** a Department of Neurobiology, School of Basic Medical Sciences, Peking University, Beijing, China; b Key Laboratory for Neuroscience, Ministry of Education/National Health Commission of China, Beijing, China; c Department of Clinical Laboratory, Beijing Chao-Yang Hospital, Capital Medical University, Beijing, China

**Keywords:** Cocaine use disorder, Dopamine D2 receptor, Protein kinase D1, Locomotor activity, Peptide

## Abstract

Locomotor hyperactivity is an early behavioural adaptation in cocaine use disorder, driven by increased dopamine levels in the striatum. The expression, sensitivity, and availability of dopamine D2 receptor (D2R) are significantly associated with cocaine use disorder. However, neither D2R agonists nor antagonists are optimal for clinical intervention because of their side effects. Therefore, targeting regulatory proteins that can effectively disrupt cocaine-induced D2R malfunction may offer improved strategies for cocaine use disorder. Here, we report that knockdown of protein kinase D1 (PKD1) in the rat dorsal striatum attenuates cocaine-induced locomotor hyperactivity. PKD1 phosphorylates the serine 365 site (S365) of D2R, reduces its surface localisation, and enhances downstream extracellular signal-regulated kinase (ERK) signalling. Tat-S365, an engineered Tat fusion-peptide blocked S365 phosphorylation in D2R, thereby decreasing the pERK levels. *In vivo* injection of peptide Tat-S365 into the rat dorsal striatum successfully inhibited cocaine-induced locomotor hyperactivity. Thus, targeting S365 of D2R presents a promising strategy for developing pharmacotherapeutic treatments for cocaine sensitisation and other disorders that result from dopamine imbalances.

## Introduction

Cocaine is a highly abused psychostimulant in humans that can lead to cocaine use disorder, characterised by compulsive drug use. With increasing wealth and societal demands, cocaine use has become more prevalent (Orson et al., 2014). However, effective treatment options for cocaine use disorder remain elusive (Shorter and Kosten, 2011). Therefore, it is crucial to comprehend the neurobiological mechanisms underlying cocaine use disorder and identify potential targets for pharmacotherapeutic intervention.

Protein kinase D1 (PKD1) belongs to the serine/threonine protein kinase family, consisting of three members (Czöndör et al., 2009; Rey et al., 2003). PKD1 is implicated in neuronal development (Czöndör et al., 2009; Cen et al., 2018), neurotransmission (Arkhipov and Pavlov, 2019; Carotti et al., 2022), drug use disorder (Wang et al., 2014), and learning and memory (Wang et al., 2014, 2018, 2022). We previously reported that the phosphorylation of dopamine D1-like receptors (D1R) by PKD1 in the hippocampus and ventral striatum promotes context-induced cocaine reward learning, while PKD1 in dorsal striatum contributes to cocaine-induced locomotor hyperactivity (Wang et al., 2014). Given that the inhibition of PKD1-mediated phosphorylation of D1R in the dorsal striatum resulted in only a partial reduction in cocaine-induced hyperactivity, compared to the knockdown of PKD1, it raises the question whether PKD1 may regulate additional target molecules that contribute to cocaine-induced hyperactivity. Therefore, further investigation is necessary to fully elucidate the role of PKD1 in this process.

Dopamine exerts its functions mainly through D1- and D2-like receptors (Beaulieu and Gainetdinov, 2011; Myslivecek, 2022). Clinical and preclinical research have primarily focused on the significance of D2R in cocaine use disorder. Using imaging techniques, studies have shown reduced availability of D2R in persons with active cocaine use disorder, and at different time points during recovery, up to a year after withdrawal (Volkow et al., 1993, 2014). Previous research on mice and monkeys has also shown that chronic cocaine usage or self-administration leads to a decrease in D2R expression, sensitivity, and availability in various regions of the brain such as the striatum, caudate nucleus, and putamen (Say et al., 2022; Dai et al., 2022; Thompson et al., 2010). Additionally, reduced D2R availability can predispose animals to cocaine use, as monkeys and rats with low baseline D2R availability have shown a propensity to self-administer cocaine and display enhanced compulsive drug-seeking and cocaine use disorder (Nader et al., 2006; Everitt et al., 2008). Phosphorylation of D2R affects its desensitisation, internalisation, and downstream signalling, such as D2R arrestin-mediated signalling and G protein signalling (Jomphe et al., 2006; Namkung and Sibley, 2004; Morris et al., 2007). Phosphosite-specific antibodies provide a vital approach to validate the importance of phosphorylation of D2R in regulating downstream signalling (Mann et al., 2021). Reduced D2R activation disinhibits downstream adenylyl cyclase and cyclic adenosine monophosphate (cAMP) production *via* Gαi/o (Nishi et al., 1997; Stoof and Kebabian, 1981), phospholipase C activation (Kanterman et al., 1991). D2R agonist-induced internalisation of the D2 receptor (D2R) (Skinbjerg et al., 2009) and stimulation of D2R resulted in extracellular signal-regulated kinase1/2 (ERK) activation (Liu et al., 2023). Most importantly, D2R plays a crucial role in cocaine-induced hyperlocomotion (Volkow et al., 1993, Volkow et al., 2006; Nader et al., 2006; Dalley et al., 2007).

Locomotor hyperactivity is an early behavioral adaptation in cocaine use disorder, driven by the increase of dopamine in striatum. In the present study, we aimed to investigate whether PKD1 regulates cocaine hyperlocomotion through phosphorylation of D2R and D2R-mediated downstream signalling in dorsal striatum. We found that PKD1 phosphorylates D2R at its serine 365 site in the third intracellular loop (IL3), reduces its membrane localisation, and thereby disinhibits ERK activity. Microinjection of a cell-permeable Tat fusion-peptide targeting S365 of D2R (Tat-S365) into the dorsal striatum disrupted PKD1-mediated phosphorylation of D2R and significantly alleviated cocaine-induced locomotor hyperactivity in rats. Given the pressing need for effective disease-modifying treatments for cocaine use disorder, the identification of its molecular basis and the potential therapeutic effects of Tat-S365 in cocaine use disorder are highly valuable findings.

## Materials and methods

### Antibodies

Anti-phospho Ser/Thr antibody (mouse, SC81514) and anti-ERK1/2 antibody (rabbit, sc-93) were bought from Santa Cruz Biotechnology (Dallas, Texas, USA). Anti-phospho-ERK1/2 antibody (rabbit, 4370S) was obtained from Cell Signaling Technology (Danvers, Massachusetts, USA). Anti-β-actin antibody (mouse, A5316), anti-PKD1 antibody (rabbit, SAB4502371), anti-glyceraldehyde-3-phosphate dehydrogenase (GAPDH) (mouse, G8795), and anti-β-tubulin (mouse, T5201) were purchased from Sigma (St. Louis, Missouri, USA). Anti-green fluorescent protein (GFP) antibody (G6539) was purchased from Roche Diagnostics (Basel, Switzerland).

### Plasmid constructs

The his-myc-PKD1 and his-myc-DNPKD1 constructs were gifts from Professor Yun Wang at Peking University. His-myc-PKD1 was generated using the *Xho*I/*Eco*RI sites and subcloned into pcDNA 3.1/myc-His C (Invitrogen). His-myc-DNPKD1 (**D727A**) was created using the QuickChange site-directed mutagenesis kit (Stratagene, La Jolla, CA). Rat D2Rs were synthesised and purified using GL Biochem (Shanghai, China), and D2R was subcloned into pEGFP-N1 vector. Mutants of GFP-D2R-**S365A**, GFP-D2R-**T225A**, and GFP-D2R-**T245A** were generated using the QuickChange site-directed mutagenesis kit (Stratagene, La Jolla, CA).

### Drug and Peptides

Cabergoline (FCE-21336) was purchased from MedChemExpress (Shanghai, China). Cocaine HCl (Qinghai Pharmaceutical) was dissolved in 10 mg/ml of 0.9 % physiological saline. Go6976 (Calbiochem) (San Diego, California,USA) was dissolved in dimethyl sulfoxide (DMSO) (Calbiochem). Tat-S365 (RKKRRQRRR-LKTMSRRKL***S***QQKE) and Tat-S365A (RKKRRQRRR-LKTMSRRKL***A***QQKE) were synthesised and purified by GL Biochem (Shanghai, China). Peptides were dissolved in 10 mg/ml of ddH_2_O. The peptide was injected into the bilateral dorsal striatum of rats in 4 min (0.25 μl/min), and an additional 1 min was allowed for diffusion before the injection needle was taken out.

### Lentivirus

GFP-PKD1-shRNA lentivirus were gifts from Professor Yun Wang at Peking University. The sequence 5’-GGTTCTGGACAGTTCGGAA-3’ was connected to the U6-vshRNA-UBI-GFP (Cen et al., 2018). Lentivirus expressing a mismatch shRNA with a scrambled sequence and no known homology to a rat gene (scrambled shRNA) was used as the control (sequence: 5’-TTCTCCGAACGTGTCACGT-3’). Human PKD1 (hPKD1) full length rescue lentivirus and its GFP control were also obtained from Genechem (Shanghai, China). For *in vivo* knockdown and rescue expression of PKD1, 200 nl of 1 × 10^8^ lentiviral particles were injected bilaterally into the dorsal striatum.

### Cell culture and transfection

HEK 293 cells were cultured in Dulbecco's Modified Eagle Medium (DMEM) (Gibco-BRL, Waltham, Massachusetts, USA) containing 10 % foetal bovine serum (FBS) (Hyclone, Logan, UT, USA). The cells were cultured in an incubator at 37 °C and 5 % CO2. Cell transfection was performed with lipofectamine 3000 (Invitrogen) for 4–6 h, and the cell culture medium was then replaced. Cells were used 24–36 h post transfection.

### Cell surface biotinylation assay

HEK 293 cells were transfected with his-myc-PKD1 or his-myc-empty-vector and GFP-D2R; 24–36 h after transfection, the cells were biotinylated with 0.4 mg/ml EZ-link sulfo-NHS-LC-biotin (Pierce) in Dulbecco's phosphate-buffered saline (DPBS), containing Ca^2+^ and Mg^2+^ for 1 h at 4 °C. Then, the cells were gently washed three times with PBS containing 100 mm glycine to remove the excess biotin. The cells were lysed in radioimmunoprecipitation assay (RIPA) buffer (25 mm Tris, pH 7.4, 137 mm NaCl, 2.7 mm KCl, 1 % Triton X-100, 0.1 % sodium dodecyl sulphate (SDS), and proteinase inhibitor mixture). Cell lysates containing 200 μg proteins were incubated with Ultralink Plus immobilised streptavidin beads (Pierce) overnight at 4 °C. After being washed with RIPA buffer five times, bound proteins were eluted by boiling for 5 min and analysed using western blot (with anti-GFP antibody), to detect surface localisation of GFP-D2R. HEK 293 cells were transfected with his-myc-PKD1 or his-myc-empty-vector and GFP-D2R; 24–36 h post transfection, the cells were lysed in RIPA buffer, and 20 μg protein was boiled for 5 min and analysed using western blot (with anti-GFP antibody) to detect total GFP-D2R levels. β-actin was used as the control.

### Western blot analysis

HEK 293 cells were transfected with his-myc-empty-vector, his-myc-PKD1, or his-myc-DNPKD1, and GFP-D2R; 24–36 h later, cells were lysed in RIPA buffer (25 mm Tris, pH 7.4, 137 mm NaCl, 2.7 mm KCl, 1 % Triton X-100, 0.1 % SDS, and proteinase inhibitor mixture). The cell lysate was then centrifuged at 12,000 g for 4 min at 4 °C. The supernatant containing proteins were used for western blot with anti-P-ERK antibody and anti-GAPDH or β-tubulin antibody to detect P-ERK and GAPDH or β-tubulin levels.

### *In vitro* kinase assay

The *in vitro* kinase assay was conducted as previously described (Yang et al., 2007); 5 μl of His-fusion proteins (His-D2R-IL3, His-D2R-IL3 T225A, His-D2R-IL3 T245A, His-D2R-IL3 T365A) synthesised using Ruibo (Beijing, China) was added to a final volume of 25 μl of kinase reaction buffer containing 5 μCi [γ-^32^P] ATP, dithiothreitol (DTT), and 20 ng purified PKD1 protein (Upstate Biotechnology, Lake Placid, NY). Then the mixture was bathed at 30 °C for 30 min, and 6 × loading buffer was added to the mixture and boiled for 5 mins. The mixture was then placed on ice for 5 mins and transient centrifugation was performed. Finally, the samples were subjected to SDS-polyacrylamide gel electrophoresis (PAGE), stained with Coomassie blue, dried, and exposed to X-ray film for autoradiography.

### Immunoprecipitation

When 293 T cells attained approximately 70 % confluency, they were transfected using lipofectamine 3000 with GFP-D2R and His-myc-PKD1, or His-myc-vector; 48 h post transfection, 50 µM of either D2R interfering peptide TAT-365 or its control TAT-365A was added. Both were solubilised in PBS and incubated at 37 °C for 30 min. Following incubation, the culture medium was aspirated, the cells were washed with pre-chilled PBS, and harvested. The cells were resuspended in 300 µl of cell lysis buffer, which included a protease inhibitor cocktail, and incubated on ice for 15 min to facilitate lysis. Subsequently, the lysate was centrifuged at 14,000 *g* for 10 min at 4 °C. The supernatant was carefully transferred for further analysis. Then, 2.5 µg of GFP antibody was added to the whole cell lysate and incubated on a rotator at 4 °C overnight. Subsequently, pre-washed protein A/G agarose beads were added and incubation was continued for 4 h at 4 °C on the rotator. Centrifugation was performed at low temperature and the supernatant was carefully discarded. The centrifuged pellet was resuspended in SDS lysis buffer and the proteins were eluted by heating at 95 °C for 5 min. Following this, centrifugation was performed at 12,000 rpm for 30 s at 4 °C, and the supernatant was collected and analysed using western blotting. A serine phosphorylation-specific antibody was used to evaluate the phosphorylation level of serine.

### Animals

All animal experiments were approved by the Animal Care and Use Committee of Peking University (Approval number: BJCB0086) and were performed in accordance with relevant guidelines and regulations. Male Sprague–Dawley rats (approximately 250 g, 8–10 weeks) were maintained in a hot (23 ± 2 °C) and humid (50 ± 5 %) environment in a 12 h light/dark cycle with food and water available ad libitum. The house lights were off during the day and on in the night.

### Surgery and stereotactic injection

Male SD rats were anaesthetised with sodium pentobarbital anaesthesia, and the cannulas were implanted 1 mm above the dorsal striatum on both sides. The coordinates for the dorsal striatum were anterior/posterior + 1.0 mm, medial/lateral ± 2.5 mm, and dorsal/ ventral − 5.5 mm. At the end of the experiment, each rat was injected with 20,000 units of penicillin on its back to prevent infection. The rats were given at least 5 days to recover before the experiments.

### Locomotor activity

The measuring instrument used was the spontaneous locomotion activity assay system from Zhongshi Anilab. Four days before the measurement, male SD rats were placed in the circadian reversal rhythm, while other conditions were normal; 2 days before the measurement, rats were placed in the instrument for 1 h every day to acclimatise. The assay was performed in the dark. At the beginning of the experiment, the computer test system was started and the measurement was performed. Male SD rats were placed in the instrument for 1 h, then injected with cocaine or saline, and the locomotion activity was measured for 1 h. The measurement was set to record a movement distance value every 5 min to plot a distance time curve. In the peptides injection experiment, male SD rats were placed in the instrument for 0.5 h and then treated with Tat-S365 or Tat-S365A (control peptides). Cocaine or saline was injected 0.5 h later, and the locomotion activity of the rats was measured for 1 h.

### Statistical analysis

The data are expressed as mean ± standard error of mean (SEM). Statistical analysis was performed using Prism 5.0 software. Differences between groups were compared using Student’s *t*-test, and one-way analysis of variance (ANOVA) analysis was followed by Newman–Keuls post hoc test or two-way ANOVA analysis followed by Bonferroni’s post hoc test. Statistical significance was set at *P* < 0.05.

## Results

### PKD1 contributes to cocaine-induced locomotor hyperactivity

**Fig. 1:**
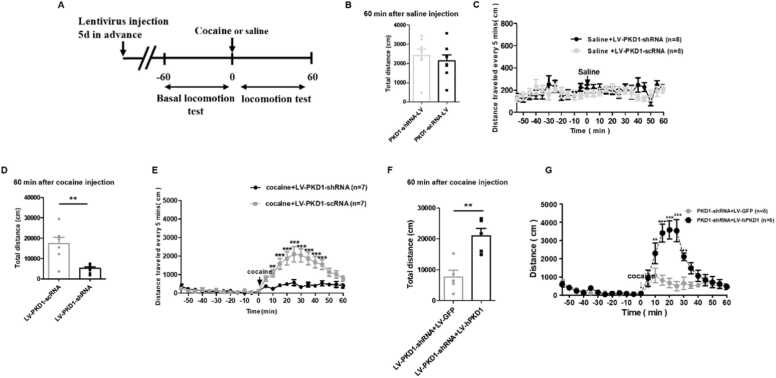
PKD1 in rats’ dorsal striatum promotes cocaine-induced locomotor activity. (A) Timeline of lentivirus injection into the bilateral dorsal striatum, cocaine or saline administration, and locomotion behaviour test. (**B**) Total distance travelled by LV-*Pkd1*-shRNA- (2424 ± 323.6 cm) or LV-*Pkd1*-scRNA- (2137 ± 310.0 cm) injected rats during 0–60 min after saline injection. (n = 8) (**C**) Distance travelled every 5 min by LV-*Pkd1*-shRNA- or LV-*Pkd1*-scRNA-injected rats before and after saline injection. (n = 8) (**D**) Total distance travelled by LV-*Pkd1*-scRNA- (17,390 ± 3050 cm) or LV-*Pkd1*-shRNA- (5090 ± 836.3 cm) injected rats during 0–60 min after cocaine injection (n = 7). (**E**) Distance travelled every 5 min by LV-*Pkd1*-shRNA- or LV-*Pkd1*-scRNA-injected rats before and after cocaine injection. (**F**) Total distance travelled by LV-PKD1-shRNA + LV- GFP (7602 ± 2175 cm) or LV-PKD1-shRNA + LV- hPKD1 (21,010 ± 2409 cm) co-injected rats during 0–60 min after cocaine injection (n = 5). (**G**) Distance travelled every 5 min by LV-PKD1-shRNA + LV-hPKD1 or LV-PKD1-shRNA + LV-GFP co-injected rats before and after cocaine injection (n = 5). Unpaired *t*-test was performed in B, D, F. **P < 0.01, cocaine +LV-PKD1-shRNA *vs.* cocaine + LV-PKD1-scRNA, LV-PKD1-shRNA + LV-hPKD1 *vs*. LV-PKD1-shRNA + LV-GFP. Two-way ANOVA followed by Dunnett's and Šídák's multiple comparisons test was performed in C, E, and G. **P < 0.01, ***P < 0.001, cocaine + LV-PKD1-shRNA *vs.* cocaine + LV-PKD1-scRNA in (E) and PKD1-shRNA + LV-GFP *vs.* PKD1-shRNA + LV-hPKD1 in (G).

Our previous study demonstrated a significant increase of PKD1 activation in the dorsal striatum upon acute cocaine injection. Bilateral microinjection of lentivirus (LV) expressing *Pkd1* shRNA significantly knocked down the expression of PKD1 in the dorsal striatum (Wang et al., 2014). Lentivirus expressing scrambled RNA (LV-*Pkd1*-scRNA) sequence was used as the control. A single intraperitoneal (*i.p*) injection of cocaine (20 mg/kg) or the same volume of saline was used (Fig. 1A). The results showed that compared to scrambled shRNA, bilateral microinjection of LV-*Pkd1*-shRNA into the dorsal striatum did not affect the distance travelled by the rats when they were treated with saline (Fig. 1B–C). A single injection of cocaine induced the rats to travel a significantly longer distance, and this effect lasted at least 60 min after the injection. Compared to *LV-Pkd1-scRNA*, bilateral microinjection of LV-*Pkd1*-shRNA into the dorsal striatum significantly reduced the distance travelled by cocaine-injected rats (Fig. 1D–E). To further verify whether this blockade of cocaine behaviour response is specific to dorsal striatum PKD1 knockdown, we performed a hPKD1 rescue experiment. The effect of LV-hPKD1 in reversing LV-*Pkd1*-shRNA knockdown has been previously validated (Cen et al., 2018). Results showed that, compared to LV-Pkd1-shRNA and LV-GFP co-injection, co-injection of LV-Pkd1-shRNA and full-length hPKD1-expressing lentivirus significantly enhanced the rats’ locomotion, and cocaine induced these LV-hPkd1-injected rats to travel longer for at least 40 min post injection (Fig. 1F–G). The abovementioned results suggest that dorsal striatum PKD1 is required for cocaine-induced locomotor hyperactivity but not general locomotion of rats.

### PKD1 inhibits D2R surface localisation and reduces its function

**Fig. 2:**
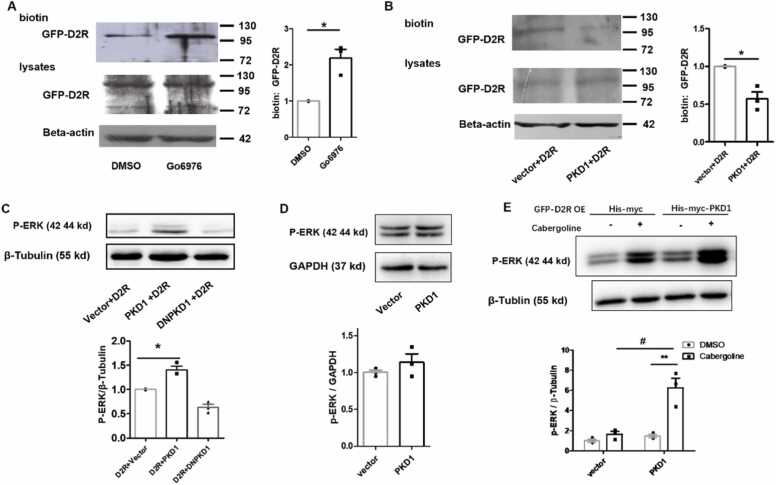
PKD1 reduces D2R surface expression and disinhibits downstream ERK signalling. (A) Surface localisation and total expression of GFP-D2R in HEK293 cells transfected with GFP-D2R and his-myc-PKD1, and subsequent treatment of HEK 293 cells with PKD1 inhibitor Go6976 (1μM) or its DMSO vehicle (n = 3). inhibition of PKD1 significantly improved GFP-D2R surface localisation. *P < 0.05. **(B)** Surface localisation and total expression of GFP-D2R in HEK293 cells transfected with GFP-D2R and his-myc-empty-vector or his-myc-PKD1 (n = 3). PKD1 overexpression significantly reduced its surface localisation**.** *P < 0.05**. (C)** Transfection of his-myc-PKD1 but not his-myc-DNPKD1 or his-myc-empty-vector increased ERK phosphorylation in HEK293 cells transfected with GFP-D2R (n = 3). *P < 0.05. **(D)** Compared to his-myc-empty-vector, PKD1 did not affect the phosphorylation of ERK in HEK293 cells transfected with his-myc-PKD1 alone (n = 3). **(E)** HEK 293 cells were transfected with GFP-D2R and his-myc-vector or his-myc-PKD1, and treated with 10 μM cabergoline or DMSO for 24 h. When cabergoline was applied, compared to his-myc-vector, PKD1 overexpression significantly improved the phosphorylation of ERK. #*P* < 0.05. In HEK 293 cells co-transfected GFP-D2R and his-myc-PKD1, compared to DMSO, cabergoline greatly enhanced the phosphorylation of ERK (n = 3). **P < 0.01. Paired *t*-test was performed as shown in A–E.

Since PKD1 is a serine/threonine protein kinase that phosphorylates its targets and regulates their function, the question arose whether PKD1 participated in cocaine-induced locomotor activity through its regulation of D2R. HEK 293 cells were transfected with his-myc-PKD1 and GFP-D2R, and PKD1 was inhibited with the inhibitor Go6976 (1 μM) at 6 h after transfection. Surface biotinylating assay and western blot were performed 24 h after transfection to detect the total (lysates) protein level and surface protein level of GFP-D2R. The results showed that inhibition of PKD1 did not affect the total protein level of GFP-D2R but significantly improved GFP-D2R surface localisation (Fig. 2A). As Go6976 is not specific for PKD1, HEK 293 cells were transiently transfected with GFP-D2R and his-myc-empty-vector or his-myc-PKD1, and surface biotinylating assay and western blot were performed to detect the total (lysates) protein level and surface protein level of GFP-D2R. Results showed that co-transfection of his-myc-PKD1 did not affect the total protein level of D2R but significantly reduced its surface localisation (Fig. 2B). These data indicate that PKD1 could regulate the surface localisation of D2R.

D2R agonist induced internalisation of the D2 receptor resulting in upregulation of downstream ERK signalling (Liu et al., 2023). To test whether PKD1 affected downstream ERK signalling, the level of ERK1/2 expression and activation in HEK 293 cells transfected with GFP-D2R and his-myc-PKD1 were examined. Results showed that overexpression of PKD1 could significantly elevate the phosphorylation of ERK1/2 (pERK). Neither his-myc-empty-vector nor his-myc-DNPKD1 transfection altered the phosphorylation of ERK1/2 (Fig. 2C). HEK 293 cells were then transfected with his-myc-vector or his-myc-PKD1, and the results showed that without D2R overexpression, PKD1 did not affect the phosphorylation of ERK1/2 (Fig. 2D). HEK 293 cells were transfected with GFP-D2R and his-myc-vector or his-myc-PKD1 and treated with 10 μM D2R agonist cabergoline or DMSO; 24 h later, western blot results showed that, compared to his-myc-vector, when PKD1 was provided, cabergoline greatly enhanced the phosphorylation of ERK. We also found that, in HEK 293 cells co-transfected with GFP-D2R and his-myc-PKD1, compared to control DMSO, D2R agonist cabergoline significantly enhanced the phosphorylation of ERK (Fig. 2E).

### Blocking PKD1-mediated phosphorylation on D2R inhibits ERK activation

**Fig. 3:**
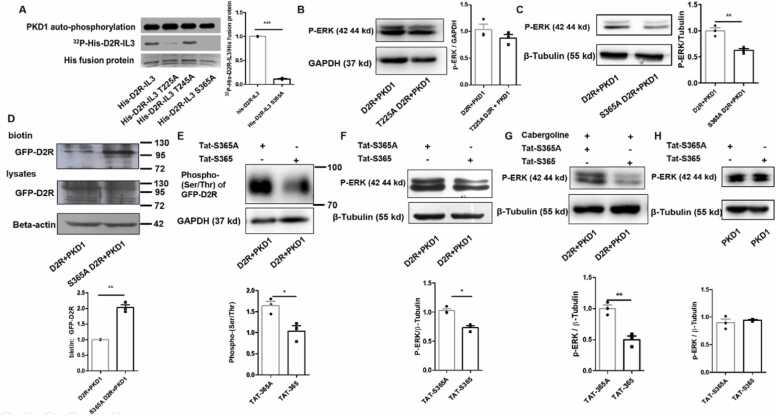
Blocking PKD1-mediated phosphorylation of D2R serine 365 site inhibits ERK activation. (A) *In vitro* kinase assay revealed that purified PKD1 phosphorylates the third intracellular loop of D2R (D2R-IL3) in WT group. D2R site mutation at T225 (T225A) significantly attenuated this phosphorylation and D2R site mutation at S365 (S365A) completely abolished this phosphorylation. T245A mutation had no effect on PKD1-mediated D2R phosphorylation (n = 3). **(B)** Transfection of HEK 293 cells with his-myc-PKD1 and GFP-D2R or GFP-D2R T225A, T225 mutated to A, did not affect the phosphorylation of ERK (n = 3). **(C)**Transfection of PKD1-expressing vector failed to alter the upregulated phosphorylation of ERK in HEK293 cells transfected with S365A mutated GFP-D2R (n = 3). **P < 0.01. (D) HEK 293 cells co-transfected with his-myc-PKD1 and GFP-D2R or GFP-D2R S365A and GFP-D2R S365A significantly improved GFP-D2R surface localisation (n = 3). **P < 0.01 **(E)** HEK 293 cells were co-transfected with his-myc-PKD1 and GFP-D2R and treated with Tat-S365 or Tat-S365A after 48 h. In the subsequent IP experiments, GFP antibody was used to precipitate GFP-D2R protein. Results showed that Tat fusion-peptide targeting S365 site effectively decreased the phosphorylation of Ser/Thr of GFP-D2R (n = 3). *P < 0.05. **(F)** Tat fusion-peptide targeting S365 site effectively decreased the phosphorylation of ERK in GFP-D2R and his-myc-PKD1 transfected HEK 293 cells (n = 3). *P < 0.05. **(G)** When 10 μM cabergoline (D2R agonists) was applied for 24 h, Tat fusion-peptide targeting S365 site effectively decreased the phosphorylation of ERK in GFP-D2R and his-myc-PKD1 transfected HEK 293 cells, (n = 3). **P < 0.01. **(H)** Compared to Tat-S365A, Tat-S365 did not affect the phosphorylation of ERK in HEK293 cells transfected with his-myc-PKD1 alone (n = 3). Paired *t*-test was performed as shown in A–G, *P < 0.05, **P < 0.01, ***P < 0.001.

To test whether the abovementioned regulation is mediated by direct phosphorylation, we found two potential PKD1 phosphorylation sites (*I/L/V*-X-*K/R*-X-X-S/T) (Rozengurt, 2011) in D2R, threonine 225 (T225) and serine 365 (S365), both of which are located within IL3 of D2R. An *in vitro* kinase assay demonstrated direct phosphorylation of IL3 of D2R by PKD1. Mutation of T225 to alanine (T225A) reduced the phosphorylation, and mutation of S365 to alanine (S365A) completely abolished the phosphorylation (Fig. 3A). However, phosphorylation remained unaffected when T245, which is not the PKD1 phosphorylation site, was mutated to alanine (T245A) (Fig. 3A). These results suggest that PKD1 phosphorylates D2R within IL3 at T225 and S365, but not T245.

PKD1-mediated increase in ERK phosphorylation was not affected by S225A mutation of D2R (Fig. 3B). But PKD1-mediated increase in ERK phosphorylation was abolished by S365A mutation in D2R, and p-ERK levels were comparable between D2R and S365A D2R groups, showing that overexpression of PKD1 could no longer alter p-ERK levels if S365 site is mutated (Fig. 3C). These data indicate that the S365 site of D2R is involved in the regulatory effect of PKD1 on D2R-mediated ERK signalling. To investigate whether phosphorylation of S365 in D2R affected the surface localisation of D2R, HEK 293 cells were co-transfected with his-myc-PKD1 and GFP-D2R or GFP-D2R S365A; surface biotinylating assay and western blot showed that when S365 was mutated to alanine, surface localisation of D2R was notably improved (Fig. 3D). Tat-mediated intracellular delivery of functional peptides is an effective intervention method that has been proved by us and others (Cen et al., 2018; Wang et al., 2014; Li et al., 2014). Thus, we constructed a Tat-S365 peptide to inhibit the phosphorylation of D2R at the S365 site. HEK 293 cells were transfected with GFP-D2R and his-myc-PKD1, and 48 h later, the cells were treated with Tat-S365 or Tat-S365A. GFP antibody was used to precipitate GFP-D2R. Western blot results showed that Tat-S365 could effectively reduce the Ser/Thr phosphorylation of D2R (Fig. 3E). In HEK 293 cells co-transfected with his-myc-PKD1 and GFP-D2R, we found that Tat-S365 also reduced the phosphorylation of ERK (Fig. 3F). When D2R agonist cabergoline was applied, we found that in HEK 293 cells transfected with GFP-D2R and his-myc-PKD1, Tat-S365 could significantly reduce the phosphorylation of ERK, compared to Tat-S365A (Fig. 3G). But compared to Tat-S365A, Tat-S365 could not affect the phosphorylation of ERK when HEK 293 cells were only transfected with his-myc-PKD1 (Fig. 3H), indicating that Tat-S365 may be efficient in blocking PKD1-mediated D2R phosphorylation, thereby inhibiting downstream ERK activation.

### Attenuation of cocaine-induced locomotor hyperactivity by the Tat-S365 peptide

**Fig. 4:**
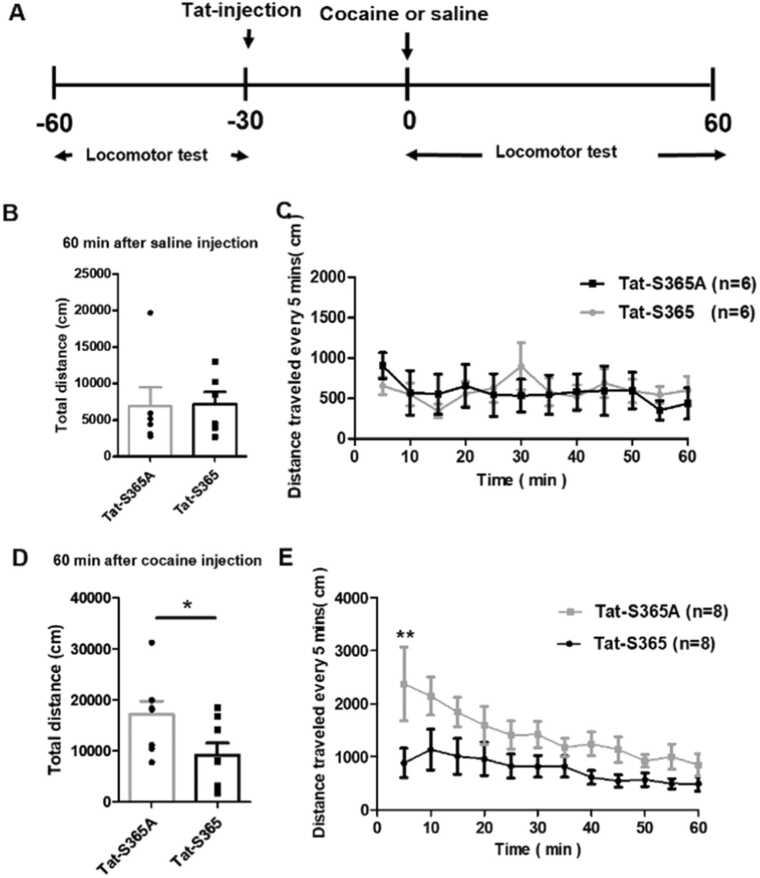
Attenuation of cocaine-induced locomotor hyperactivity by Tat-S365. (A) Timeline of the bilateral Tat-peptide microinjection into the dorsal striatum and locomotion behaviour test. (**B**) Tat-S365 (7141 ± 1659 cm) and Tat-S365A (6855 ± 2613 cm) were injected into the dorsal striatum 30 mins before saline injection (n = 6). Total distance travelled during 0–60 min after saline injection. (**C**) Distance travelled by rats every 5 min after saline injection. (**D**) Total distance travelled by Tat-S365-injected (9164 ± 2307 cm) or Tat-S365A-injected (17,110 ± 2626 cm) rats after cocaine injection (n = 8). (**E**) Distance travelled every 5 min by Tat-S365-injected or Tat-S365A -injected rats after cocaine injection (n = 8). Students’ *t*-test was performed as shown in **B, D,** *P < 0.05. Two-way ANOVA followed by Šídák's multiple comparisons test was performed as shown in C and E. **P < 0.01 Tat-S365 *vs.* Tat-S365A.

Given that knockdown of PKD1 could effectively attenuate cocaine-induced locomotor hyperactivity, and Tat-S365 was effective in blocking PKD1-mediated ERK activation, we wondered whether Tat-S365 could also attenuate cocaine-induced locomotor hyperactivity. Tat-S365 was microinjected into the bilateral dorsal striatum of rats, and their locomotion was observed following cocaine or saline injection. Results showed that neither Tat-S365 peptide nor its negative control (Tat-365A) altered the locomotor activity of rats when saline was injected. No significant change was observed in total distance travelled over a period of 60 min (Fig. 4B) or distance travelled every 5 min (Fig. 4C). A single injection of cocaine still induced the Tat-365A-injected rats to travel longer, both when analysed every 5 min or over the duration of 60 min, whereas cocaine had almost no effect on Tat-365-injected rats (Fig. 4D–E). Together, these data indicate that Tat-S365 could successfully attenuate cocaine-induced locomotor hyperactivity.

## Discussion

Dopamine interacts with dopamine receptors and improves the activation of phospholipase C (PLC), resulting in the production of a lipid second messenger diacylglycerol (DG) from the hydrolysis of phosphatidylinositol 4,5- bisphosphate. DG is known to mediate a number of cascades through the DG-binding C1 domain of various proteins, including protein kinase C (PKC) (Nishizuka, 1992), as well as PKD (Baron and Malhotra, 2002). PKC inhibitors could suppress cocaine-mediated behaviours (Cervo et al., 1997). However, the activation of PKC lead to PKD activation by phosphorylating serine at positions 744 and 748. PKC inhibitors GF109203X, Go6976, and Go6983 inhibited PKD1, S744, and 748 by 87 %, 13 %, and 82 %, respectively (Berna et al., 2007). Abdominal injection of cocaine in rats significantly enhanced phosphorylation of PKD1 S744 and 748 in the striatum (Wang et al., 2014). Lentivirus-mediated PKD1 knockdown in the dorsal striatum significantly reduced cocaine-induced locomotor hyperactivity, indicating that PKD1 activity is essential for cocaine-induced locomotor hyperactivity and can increase this hyperactivity through unregular D1R surface expression and phosphorylation. Peptide was used to disturb the phosphorylation of D1R by PKD1, leading to reduced cocaine-induced locomotor hyperactivity (Wang et al., 2014). However, cocaine-induced locomotor hyperactivity of rats treated with the peptide were much higher than that in rats treated with PKD1-shRNA, indicating that pathways other than D1R were involved. In most cases, PKD1 phosphorylates a sequence characterised by L/V/I at position-5 and R/K at position-3 (Rozengurt, 2011). However, we found that PKD1 can phosphorylate D2R at 225 threonine (the -5 position is R) and 365 serine (the -5 position is S). This inconsistency may explain why D2R has not been identified as a PKD1 substrate in previous studies.

Phosphorylation is an important post-translational modification of D2R, especially the serine/threonine phosphorylation sites within the IL3 of D2R. G protein-coupled receptor kinases 2 and 3 (GRK2 and GRK3) can phosphorylate D2R by targeting eight serine/threonine residues in IL3 (Kim et al., 2001; Namkung et al., 2009). PKC also can mediate the phosphorylation, desensitisation, and internalisation of D2 dopamine receptors. Internalisation of D2R caused by phosphorylation of D2R is β-arrestin/dynamin-dependent and is mediated through a clathrin-coated pit pathway (Namkung and Sibley, 2004). When GPCR was phosphorylated by GRK, the phosphorylation affected the conformation of GPCR and its interaction with arrestin-2 (Guillien et al., 2023). Phosphorylation sites in IL3 and C-terminus were essential for the formation of a stable complex of GPCR and β-arrestin (Huang et al., 2020). Above all, phosphorylation of D2R is very important for D2R cell membrane localisation; the question is if there are any other sites of phosphorylation that can also lead to changes in D2R cell membrane localisation. Here, we provide evidence for the involvement of PKD1 in D2R S365 phosphorylation and the consequent reduction of its surface localisation.

Our *in vitro* kinase assay (Fig. 3) revealed that mutation at T225 alone impaired the PKD1-induced phosphorylation level of D2R-IL3, with some residual phosphorylation mediated by other sites. Interestingly, mutation at S365 ablated almost all phosphorylation, with no trace of T225-mediated residual phosphorylation left. It appears that S365A also affected T225 phosphorylation. Since previous studies reported that phosphorylation of GPCR could affect its conformation (Guillien et al., 2023), our results suggest that phosphorylation at S365 might change the conformation of D2R-IL3 and expose the T225 site, enabling it to be phosphorylated. Once S365A occurred, neither site could be phosphorylated. In addition, given that mutation at T225A did not affect the intracellular pERK level, we focused our research on the D2R S365 site thereafter.

Dopamine D2R has two isoforms: short and long isoforms (dopamine D2S and dopamine D2L receptors). D2_S_R and D2_L_R are only different in the additional 29 amino acids present in IL3 of the dopamine D2L receptor (Morris et al., 2007). Dopamine D2_S_R is expressed pre-synaptically and functions as a key negative regulator of the entire dopamine system. D2R-mediated postsynaptic effects and their cooperative/synergistic activity with D1R are probably mediated by D2_L_R (Usiello et al., 2000). Our previous results showed that PKD1 modulates the dopamine signalling pathway by phosphorylating D1R in the striatum (Wang et al., 2014). Therefore, we speculated that PKD1 could also modulate dopamine signalling caused by cocaine though D2_L_R.

D2-class dopamine receptors (D2–D4) are coupled to Gi/o, resulting in a decrease in ERK activity (Missale et al., 1998), and expression of locomotor sensitisation after cocaine administration is ERK-dependent (Fasano et al., 2009; Brami-Cherrier et al., 2005; Ferguson et al., 2006; Miller and Marshall, 2005; Valjent et al., 2006). The binding of ligand could change the conformation of GPCR, inducing intracellular signalling pathway through coupling to specific intracellular proteins (Al-Zoubi et al., 2019). The phosphorylation of GPCRs mediated the internalisation of GPCR, and reduced the receptor’s response to ligand stimulation (Jin et al., 1999). Here, we identified S365 as a critical site in surface localisation of D2R. The single site mutation of S365A impaired DA-induced ERK1/2 activation.

Although all biochemical experiments were performed in artificial and *in vitro* systems, we validated through *in vivo* experiments that disrupting D2R phosphorylation by PKD1 indeed modulates cocaine-induced increase in locomotor activity. However, further *in vivo* studies are needed to fully elucidate the role of PKD1 in cocaine-mediated behavioural responses.

**Fig. 5:**
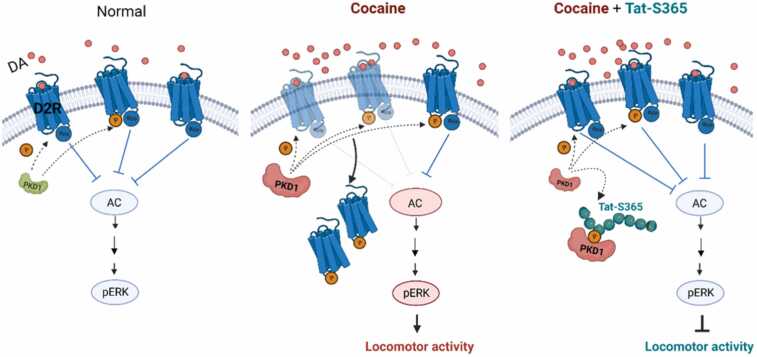
Working model. Cocaine administration-induced PKD1 activation in the dorsal striatum. PKD1 phosphorylated the serine 365 site of D2R and decreased its surface localisation, thereby enhancing downstream ERK signalling and cocaine-induced locomotor hyperactivity. Tat-S365 effectively blocked PKD1-mediated D2R phosphorylation at S365, thus maintaining D2R surface localisation, leading to attenuated cocaine-induced hyperactivity.

Taken together, our studies reveal that PKD1 is involved in cocaine-induced behavioural responses. The underlying mechanisms might be related to its reduction of surface localisation of D2R through phosphorylating S365 in the IL3. Importantly, we constructed a cell-permeable peptide, the Tat-S365 peptide, which inhibited cocaine-induced locomotor hyperactivity (Fig. 5, working model). Thus, S365 of D2R is potentially a promising candidate for pharmacotherapeutic intervention of dopamine signalling in treating drug use disorder.

## Author contribution

Linlin Sun and Ning Wang were responsible for the study concept and design. Ning Wang, Xinyu Zhang and Ziran Zhang contributed to the acquisition of data. Ziran Zhang, Ying Wang and Xinyu Zhang assisted with data analysis and interpretation of findings. Ning Wang and Linlin Sun prepared the figures. Ning Wang drafted the manuscript. Linlin Sun, Ying Wang and Xinyu Zhang supervised all experiments. All authors critically reviewed the content and approved the final version for publication.

## CRediT authorship contribution statement

**Ning Wang:** Writing – review & editing, Writing – original draft, Supervision, Project administration, Funding acquisition, Conceptualization. **Linlin Sun:** Writing – review & editing, Writing – original draft, Supervision, Funding acquisition, Data curation. **Ying Wang:** Data curation. **Ziran Zhang:** Visualization, Data curation. **Xinyu Zhang:** Validation, Data curation.

## Compliance with ethical standards

All animal experiments were approved by the Animal Care and Use Committee of Peking University (Approval number: BJCB0086).

## Funding

This work was supported by 10.13039/501100001809National Natural Science Foundation of China Grants 81901350 (N.W.) and startup funding by Peking University Health Science Center 71013Y2145 (L.S.).

## Conflicts of Interest

*The authors declare that the research was conducted in the absence of any commercial or financial relationships that could be construed as a potential conflict of interest.*

## Data Availability

Correspondence and requests for materials should be addressed to Ning Wang, Linlin Sun.
